# Spinal neurons that contain gastrin-releasing peptide seldom express Fos or phosphorylate extracellular signal-regulated kinases in response to intradermal chloroquine

**DOI:** 10.1177/1744806916649602

**Published:** 2016-06-07

**Authors:** Andrew M Bell, Maria Gutierrez-Mecinas, Erika Polgár, Andrew J Todd

**Affiliations:** 1 Institute of Neuroscience and Psychology, College of Medical, Veterinary and Life Sciences, University of Glasgow, Glasgow, UK; 2 School of Veterinary Medicine, College of Medical, Veterinary and Life Sciences, University of Glasgow, Glasgow, UK

**Keywords:** Itch, spinal cord, pERK, interneuron

## Abstract

**Background:**

Gastrin-releasing peptide (GRP) is thought to play a role in the itch evoked by intradermal injection of chloroquine. Although some early studies suggested that GRP was expressed in pruriceptive primary afferents, it is now thought that GRP in the spinal cord is derived mainly from a population of excitatory interneurons in lamina II, and it has been suggested that these are involved in the itch pathway. To test this hypothesis, we used the transcription factor Fos and phosphorylation of extracellular signal-regulated kinases (ERK) to look for evidence that interneurons expressing GRP were activated following intradermal injection of chloroquine into the calf, in mice that express enhanced green fluorescent protein (EGFP) in these cells.

**Results:**

Injection of chloroquine resulted in numerous Fos- or phospho-ERK (pERK) positive cells in the somatotopically appropriate part of the superficial dorsal horn. The proportion of all neurons in this region that showed Fos or pERK was 18% and 21%, respectively. However, among the GRP–EGFP, only 7% were Fos-positive and 3% were pERK-positive. As such, GRP–EGFP cells were significantly less likely than other neurons to express Fos or to phosphorylate ERK.

**Conclusions:**

Both expression of Fos and phosphorylation of ERK can be used to identify dorsal horn neurons activated by chloroquine injection. However, these results do not support the hypothesis that interneurons expressing GRP are critical components in the itch pathway.

## Background

Itch has been defined as an unpleasant sensation that causes a desire to scratch. Chronic itch (pruritus) can result from several dermatological and systemic diseases and represents a major unmet clinical need. Despite this, we still know relatively little about the neuronal circuits that are responsible for the sensation of itch.^1–4^ A major distinction can be made between those forms of itch that are relieved by antihistamines (histamine-dependent) and those that are not (histamine-independent). Many substances (pruritogens) can evoke itch, when injected into the skin, and these can operate through either histamine-dependent or histamine-independent mechanisms.^5–7^

An early insight into the peripheral and spinal pathways responsible for itch came from the observation that mice lacking the receptor for gastrin-releasing peptide (GRP) showed significantly reduced responses to certain pruritogens, but normal responses to a variety of painful stimuli.^8^ Three lines of evidence suggested that GRP signalling at the spinal level was required for itch: (a) intrathecal administration of GRP receptor (GRPR) agonists evoked scratching, while antagonists reduced scratching in response to injected pruritogens; (b) *in situ* hybridisation revealed that the GRPR was expressed by neurons in lamina I of the dorsal horn; (c) a subsequent study from the same group showed that ablation of spinal GRPR-expressing neurons with saporin conjugated to bombesin (an amphibian homologue of GRP) resulted in reduced responsiveness to a variety of pruritogens.^9^ This study also demonstrated that itch behaviours following administration of histamine-independent pruritogens such as chloroquine were substantially reduced in GRPR knockout mice, whereas histamine-dependent responses were much less affected.^9^ Further evidence for a role of spinal GRP signalling in histamine-independent itch came from the finding that a GRPR antagonist delivered directly to the spinal cord significantly reduced the responses of superficial dorsal horn neurons to intradermal chloroquine but not to intradermal histamine.^10^

There has been considerable debate concerning the source of GRP in the spinal cord. A number of studies have provided evidence that GRP is expressed by a specific subset of peptidergic primary afferents,^8,11–13^ and it has been suggested that the GRP released by these afferents acts through spinal GRPR to mediate itch.^8^ However, several other groups have failed to detect GRP mRNA in primary afferents, using a variety of methods, including in situ hybridisation, RT and real-time polymerase chain reaction, and RNA seq.^14–18^ In addition, it has been reported that the antibodies that had been used to reveal GRP in the dorsal root ganglion^8,13^ can cross react with substance P,^14,19^ which is expressed by many peptidergic primary afferents.^20,21^

mRNA for GRP has been identified in the dorsal horn^14–16,18,22,23^ and the GRP-expressing cells can be identified in a mouse line (Tg-GRP-EGFP) in which enhanced green fluorescent protein (EGFP) is expressed under control of the GRP promoter.^14,19,24^ It has recently been shown that these cells represent a specific population of excitatory interneurons in lamina II.^19,25^ Taken together with the evidence against primary afferent expression of GRP, these findings have led to the alternative suggestion that GRP released by itch-activated spinal interneurons plays an important role in histamine-independent itch.^14,24,26^ To test this hypothesis, we have looked for evidence that chloroquine can activate GRP-expressing dorsal horn interneurons. As GRP cannot be detected in the cell bodies of these neurons with immunocytochemistry,^19^ we used the Tg(GRP-EGFP) line. EGFP^+^ cells in this mouse are mainly present in lamina II,^19^ and it has been shown that >90% of these possess GRP mRNA.^14^To reveal activated neurons, we stained for the transcription factor Fos,^27^ which has been used in several previous studies,^28–32^ and for phosphorylated extracellular signal-regulated kinases (pERK).^33,34^

## Methods

All animal experiments were approved by the Ethical Review Process Applications Panel of the University of Glasgow and were performed in accordance with the European Community directive 86/609/EC and the UK Animals (Scientific Procedures) Act 1986.

### Fos induction

Six adult Tg(GRP-EGFP) mice of either sex (16–25 g; Gene Expression Nervous System Atlas [GENSAT]) were used to investigate Fos expression after intradermal injection of chloroquine or vehicle. The skin on the lateral aspect of the hindlimb was shaved on the day before stimulation, and in order to prevent scratching or biting of the injected area during the postinjection survival time (which would result in nociception-activated Fos), an Elizabethan collar (Harvard Apparatus, #72-0056) was applied at the time of shaving. Animals were briefly anaesthetised with isoflurane, and injections of either chloroquine (40 µg dissolved in 10 μl of phospate-buffered saline [PBS], *n* = 3 mice) or vehicle (10 μl PBS, *n* = 3 mice) were made into the lateral aspect of the left calf, after which the mice were allowed to recover from anaesthesia. The success of the intradermal injection was assessed by the formation of a small bleb^6,7^ in the calf skin. They were reanaesthetised with pentobarbitone (20 mg i.p.) and perfused through the left ventricle with fixative that contained 4% freshly depolymerised formaldehyde 2 h after the stimulus.

The L3 spinal segment (which contains the great majority of cells activated by these stimuli) was removed and postfixed for 2 h in the same fixative. The contralateral (right) side was notched to allow identification, and the tissue was cut into 60 µm thick transverse sections with a vibrating blade microtome (Leica VT1200). These were immersed in 50% ethanol for 30 min to enhance antibody penetration and then multiple-labelling immunofluorescence staining was performed as described previously.^19,35^ The sections were incubated for three days in the following combination of primary antibodies: mouse monoclonal antibody NeuN (Millipore; MAB377; diluted 1:500), chicken anti-EGFP (Abcam, ab13970; diluted 1:1000), and rabbit anti-Fos (Santa Cruz Biotechnology, sc-52; diluted 1:5,000). They were then incubated overnight in species-specific secondary antibodies that were raised in donkey and conjugated to Alexa 647, Alexa 488, or Rhodamine Red (Jackson Immunoresearch, West Grove, PA, USA). Secondary antibodies were diluted 1:500 (Alexa 647 and Alexa 488) or 1:100 (Rhodamine Red). All antibodies were diluted in PBS that contained 0.3% Triton X-100 and incubations were at 4°C. Following the immunocytochemical reaction, sections were stained with 4′,6-diamidino-2-phenylindole (DAPI) to reveal nuclei, mounted in antifade medium and stored at −20°C.

**Figure 1:**
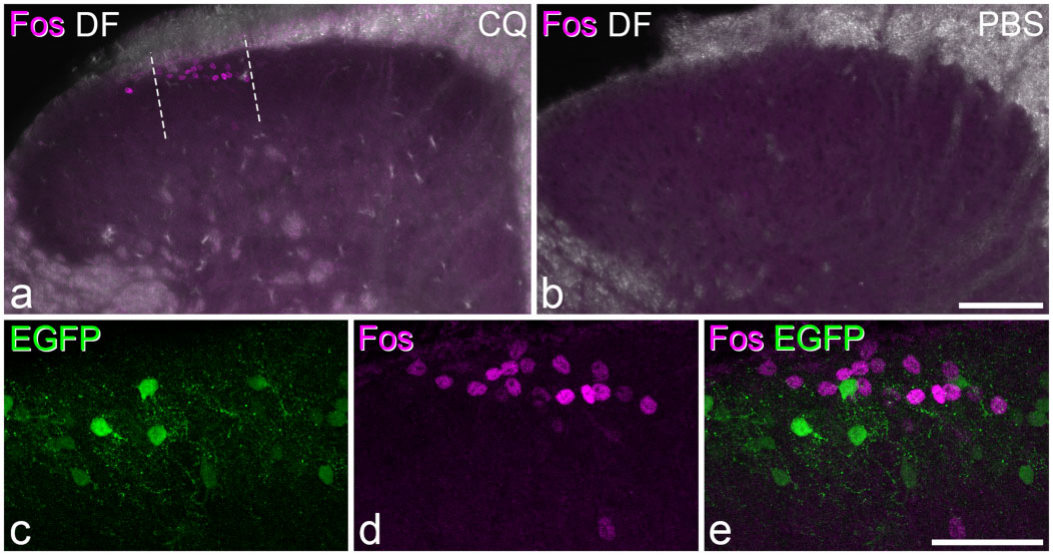
Fos in the dorsal horn following intradermal injection of chloroquine. (a) A transverse section through the dorsal horn from the L3 segment of a GRP-EGFP mouse, ipsilateral to the side in which chloroquine (CQ) had been injected intradermally 2 h prior to perfusion fixation. Fos immunoreactivity (magenta) is superimposed on a dark-field (DF) image. There is a cluster of Fos^+^ cells in the superficial part of the dorsal horn, and these are concentrated between the two dashed lines. (b) An equivalent section from a mouse that had received an intradermal injection of PBS, in which no Fos^+^ cells are visible. (c–e) Higher magnification views from the section shown in a to show the relationship between EGFP (green) and Fos. Note that although the cells are intermingled, there are none that are double labelled in this section. All sections are maximum intensity projections of confocal z-series (2 µm z-separation) through the full thickness of the 60 µm section. Scale bars: a, b = 100 µm; c–e = 50 µm.

Sections were scanned with a Zeiss LSM 710 confocal microscope equipped with Argon multiline, 405 nm diode, 561 nm solid state, and 633 nm HeNe lasers. They were initially viewed with epifluorescence optics, and three sections from the chloroquine-injected mice that contained numerous Fos^+^ cells were selected from each animal, before EGFP was observed. Z-series (2 µm spacing) were then scanned through the full thickness of each section with the 40× oil-immersion lens (numerical aperture 1.3), with the confocal aperture set to 1 Airy unit. These scans included the central part of the dorsal horn, which contained the activated cells. The z-stacks from chloroquine-injected mice were analysed with Neurolucida for Confocal (MBF Bioscience, Williston, VT, USA). The outline of the grey matter was drawn, and the ventral border of the GRP plexus (which corresponds approximately to the boundary between the inner and outer parts of lamina II^19^) was determined from a maximum intensity projection and plotted onto the drawing. The mediolateral extent of the region that contained a high density of Fos^+^ cells was delineated by drawing two parallel lines that were orthogonal to the laminar boundaries (see Figure 1).

Initially, only the channels corresponding to NeuN and DAPI were viewed, and the locations of all neurons (NeuN^+^ cells) that lay within this region were plotted onto the drawing. To avoid overcounting cells that were split during sectioning,^36^ we included cells if at least part of the nucleus (stained with DAPI) was present in the first optical section of the z-series and excluded them if part of the nucleus was present in the last optical section.^37^ The channel corresponding to Fos was then viewed, and the presence or absence of staining in each of the neurons in the sample was recorded. Finally, the EGFP channel was viewed and all neurons that were EGFP^+^ were identified on the drawing. As Fos^+^ cells were present at highest density in lamina I and the outer part of lamina II (lamina IIo), we determined the proportion of all neurons that were located within this region and between the two parallel lines that were Fos-immunoreactive. We then determined the proportion of GFP^+^ neurons within this volume that were Fos-immunoreactive. Sections from the PBS-injected mice were also examined with the confocal microscope to test for the presence of Fos-immunoreactive neurons.

### Phosphorylation of ERK

In initial studies, we performed intradermal injections of chloroquine or PBS and allowed a 5-min survival time before perfusion fixation, as phosphorylation of ERK occurs rapidly following stimulation.^33–35,38^ However, although we observed numerous pERK-positive cells in the superficial dorsal horn of the ipsilateral L3 segment in the chloroquine-injected mice, there were also many pERK^+^ cells in the corresponding region in PBS-injected mice. This is likely to have been caused by the mechanical noxious stimulus that results from needle insertion and distension of the skin during the intradermal injection.^6^ In subsequent experiments, we therefore allowed a longer postoperative survival time (30 min). Six adult Tg(GRP-EGFP) mice of either sex (16–23 g; GENSAT) were anaesthetised with isoflurane and received intradermal injections of chloroquine (40 µg in 10 μl, *n* = 3 mice) or PBS (10 μl, *n* = 3 mice) into the left calf, which had been shaved the day before, as described earlier. The mice were maintained under isoflurane anaesthesia throughout the survival period. They then received pentobarbitone (20 mg i.p.) prior to perfusion fixation (a described earlier), which was carried out 30 min after the intradermal injection. The tissue was processed exactly as described for the Fos experiments, except that the Fos antibody was replaced with rabbit anti-pERK (Cell Signaling Technology, 9101; diluted 1:500). Confocal scanning and analysis were performed as described for the Fos experiments.

### Characterisation of antibodies

The EGFP antibody was raised against recombinant full-length EGFP, and the distribution of staining matches that of native EGFP. The mouse monoclonal antibody NeuN was raised against cell nuclei extracted from mouse brain^39^ and apparently labels all neurons but no glial cells in the rat spinal dorsal horn.^40^ The Fos antibody was raised against a peptide corresponding to the *N*-terminus of human Fos. The pERK antibody detects p44 and p42 MAP kinase (Erk1 and Erk2) when these are phosphorylated either individually or dually at Thr202 and Tyr204 of Erk1 or Thr185 and Tyr187 of Erk2. It does not cross-react with the corresponding phosphorylated residues of JNK/SAPK or of p38 MAP kinase or with nonphosphorylated Erk1/2 (manufacturer’s specification). Specificity of both Fos and pERK antibodies was demonstrated by the lack of staining in nonstimulated areas (e.g. the contralateral dorsal horn).

### Statistics

Data were formatted into 2 × 2 contingency tables for each animal, with rows corresponding to presence or absence of EGFP and columns to presence or absence of Fos or pERK. To determine whether there was a consistent difference in the proportions across the tables for the different cell types, we used the Mantel–Haenszel analysis.^41^ Breslow–Day testing for homogeneity of the odds ratio was conducted prior to computation of the Mantel–Haenszel odds ratio and 95% confidence intervals.

## Results

### Fos

Injection of chloroquine into the calf, followed by a survival time of 2 h, resulted in expression of Fos in neurons in the superficial part of the dorsal horn of the L3 segment ipsilateral to the injection site. The cells were found throughout the length of the segment but were restricted to a narrow mediolateral band (around 100 µm wide) in the middle part of the dorsal horn (Figure 1a), which corresponds to the central projection zone of primary afferents from the lateral part of the calf.^42^ They were largely restricted to laminae I and IIo and were rarely seen in deeper parts of the dorsal horn. Within the delineated region, the mean total number of neurons in laminae I–IIo pooled from three sections from each animal varied from 386–440 (*n* = 3 mice), and the proportion of these that showed Fos was 18% (Table 1). In the PBS-injected mice, only very occasional cells showing Fos were evident (Figure 1b).

**Table 1:** pERK and Fos following intradermal chloroquine.

pERK/Fos	Total neurons	pERK^+^/Fos^+^ neurons	GRP-EGFP^+^ neurons	GRP-EGFP^+^ and pERK^+^/Fos^+^	% Neurons pERK^+^/Fos^+^	% GRP-EGFP neurons pERK^+^/Fos^+^
Fos	417.7 (386–440)	74 (67–78)	55 (42–62)	4.0 (1–7)	17.7 (17.4–18.3)	6.8 (2.4–11.3)
pERK	398 (309–475)	82.7 (59–110)	50 (45–60)	1.3 (1–2)	20.9 (16.6–26.8)	2.8 (1.7–4.4)

*Note*. pERK: phospho extracellular signal-regulated kinase; GRP: gastrin-releasing peptide; EGFP: enhanced green fluorescent protein. The table shows quantitative data from the region delineated by high levels of Fos or pERK, and were obtained from three mice in each case.

**Figure 2:**
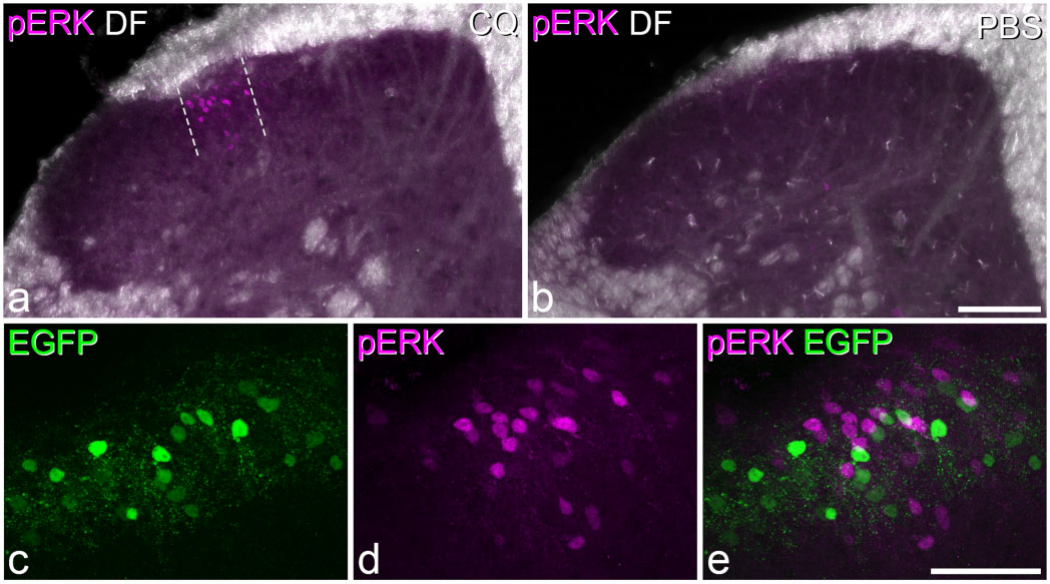
pERK in the dorsal horn following intradermal injection of chloroquine. (a) A transverse section through the dorsal horn from the L3 segment of a GRP-EGFP mouse, ipsilateral to the side in which chloroquine (CQ) had been injected intradermally 30 min prior to perfusion fixation. pERK immunoreactivity (magenta) is superimposed on a dark-field (DF) image. There is a cluster of pERK^+^ cells in the superficial dorsal horn, which is concentrated between the two dashed lines. (b) An equivalent section from a mouse that had received an intradermal injection of PBS, in which no pERK^+^ cells are visible in the superficial laminae. (c–e) Higher magnification views from the section illustrated in a show the relationship between EGFP (green) and pERK. Note that although the cells are intermingled, there are none that are double labelled in this section. All sections are maximum intensity projections of confocal z-series (2 µm z-separation) through the full thickness of the 60 µm section. Scale bars: a, b = 100 µm; c–e = 50 µm.

As reported previously,^14,19,24,25^ GRP-EGFP neurons were particularly numerous in lamina II, but were occasionally seen in lamina I, and were also scattered through the deeper parts of the dorsal horn (Figures 1c,e and 2). Although there was considerable overlap in the distribution of GRP-EGFP and Fos, relatively few of the GRP-EGFP cells were Fos-immunoreactive (Figure 1). The mean number of GRP-EGFP cells that were sampled in lamina I-IIo in the delineated area was 55, and of these, 7% were Fos-immunoreactive (Table 1).

To determine whether the presence of Fos among GRP-EGFP cells was significantly less frequent than that in the general population of neurons, we measured the common odds ratio for the three mice (Table 2). The 95% confidence interval was below 1, indicating that the GRP-EGFP cells were significantly less likely than other neurons in this region to express Fos.

**Table 2:** Analysis of odds ratios for coexpression of EGFP and activity-dependent markers.

Activity-dependent marker	Breslow–Day significance	Mantel–Haenszel test of conditional independence			Estimate of common odds ratio	95% confidence interval
		χ^2^_MH_	*df*	Significance		
Fos	0.259	13.92	1	<0.001	0.32	0.17–0.59
pERK	0.937	32.56	1	<0.001	0.09	0.03–0.25

### pERK

Thirty minutes after chloroquine injection, numerous pERK-positive cells were seen in the ipsilateral L3 segment, while in contrast, very few pERK cells were seen in the mice that had received injection of PBS (Figure 2). The distribution of pERK cells in the chloroquine-injected mice was similar to that of Fos-positive neurons described earlier, forming a narrow mediolateral band in the middle part of the dorsal horn throughout the L3 segment. The total number of neurons in laminae I-IIo within the region delineated by pERK-immunoreactivity varied from 309 to 475 (*n* = 3 mice), and the proportion of these that were pERK-immunoreactive was 21% (Table 1). Again, although numerous GRP^+^ cells were present in this region (mean 50 cells), relatively few of these (3%) were pERK-immunoreactive (Figure 2c–e). The 95% confidence interval for the common odds ratio was below 1, indicating that GRP-EGFP cells were significantly less likely than other neurons to have phosphorylated ERK (Table 2).

## Discussion

The main findings of this study are that following intradermal injection of 40 µg chloroquine into the calf, around 20% of neurons in laminae I-IIo in the somatotopically appropriate region of L3 upregulate Fos and phosphorylate ERK. However, although the activated cells showed an overlapping distribution with GRP-EGFP neurons, the latter were seldom Fos- or pERK-positive.

### Fos and pERK as markers for itch activation

Several previous studies have used expression of Fos to identify neurons in the spinal dorsal horn that were activated by various pruritogens, including chloroquine, or in models of chronic itch.^30,32,34,43–50^ Between them, these studies have involved intradermal injections into several body regions: the cheek, neck, back, calf, and hindpaw. In each case, Fos^+^ neurons have been identified in laminae I–II of the corresponding spinal cord segments or spinal trigeminal nucleus. The distribution of Fos^+^ cells seen in the present study was therefore entirely consistent with these reports. As very few Fos cells were seen following an equivalent injection of the vehicle (PBS), it is highly likely that the Fos was induced as a result of the chloroquine and therefore represents the response to a pruritic stimulus.

There have apparently been very few reports of ERK phosphorylation in itch models. Zhang et al.^34^ showed that histamine injected intradermally in the neck or hindpaw caused phosphorylation of ERK, which could be detected in the superficial dorsal horn, peaking 30 min after the stimulus. However, they found that intradermal injections of chloroquine that were sufficient to induce scratching did not evoke pERK. It is difficult to explain the discrepancy between their findings and those reported here, although in their study, the mice were not anaesthetised, and it is therefore possible that scratching of the affected area or activity in descending systems that are inactive during general anaesthesia suppressed ERK phosphorylation. It is unlikely that differences in the site of injection (neck in Zhang et al. and calf in the present study) were responsible for the different results, as Fos studies have shown very similar patterns of expression when pruritogens were injected into different sites. We found very little pERK in animals that had survived 30 min after PBS injection, which suggests that the phosphorylation was evoked by the chloroquine and therefore represents a pruritic response. However, our preliminary experiments with 5-min survival times indicated that the injection itself could cause significant phosphorylation of ERK. This was clearly very short lived, as it had completely subsided by 30 min.

Comparing our findings with Fos and pERK shows that there was a very similar distribution of labelled cells and that a comparable proportion of lamina I-IIo neurons within the somatotopically appropriate region was affected (∼20% in each case). Since ERK phosphorylation is an upstream regulator of Fos in the dorsal horn,^51^ it is likely that the two markers were labelling equivalent populations of neurons, and the finding that GRP-EGFP cells were underrepresented with both markers is consistent with this suggestion.

Interestingly, Zhang et al.^34^ reported that blocking phosphorylation of ERK with a MEK inhibitor reduced scratching behaviour in response to histamine but not to chloroquine. Our findings indicate that ERK is phosphorylated following intradermal injection of chloroquine but presumably it is not required for the resulting behaviour.

The identity of the itch-activated neurons in laminae I-IIo is not yet known, although it is likely that the great majority are interneurons,^28^ and it will therefore be important for future studies to determine which neurochemical classes of interneuron show Fos or pERK following pruritogen injections. We have recently provided evidence that four nonoverlapping populations, defined by expression of GRP, neurotensin, neurokinin B, and substance P, can be identified among the excitatory interneurons in laminae I–III.^19,25^ However, the neurotensin and neurokinin B populations are both concentrated in the inner part of lamina II and lamina III and are therefore unlikely to be involved in itch. At present, the substance P-expressing cells are difficult to identify with immunocytochemistry due to the low level of peptide present in their cell bodies.

### A role for GRP-expressing interneurons in chloroquine-evoked itch?

There is considerable evidence that the GRPR plays an important role in several forms of itch,^1^ including that evoked by chloroquine.^8,10^ As it has been suggested that GRP-expressing dorsal horn interneurons are part of the itch pathway,^14,24,26^ we were surprised to find that GRP-EGFP cells were underrepresented among those that showed either Fos or pERK. It is likely that the dose of chloroquine we used was sufficient to cause itching, as similar doses have evoked scratching or biting in other studies in the mouse.^52,53^ In addition, we have found that intradermal injection of 40 µg of chloroquine into the calf in mice without Elizabethan collars leads to biting of the injected area (EP unpublished data), and this dose was sufficient to evoke Fos or pERK in a substantial proportion (∼20%) of the neurons in laminae I–IIo in this study.

This leaves several possible explanations for this paradox. (1) A significant number of GRP-EGFP cells may have been activated without expressing Fos or phosphorylating ERK, and the risk of false-negative results should always be considered with studies involving these activity-dependent markers. It is also possible that a relatively low level of activation is required to induce release of neuropeptides, including GRP. (2) It may be that sufficient GRP is released from the relatively few GRP-EGFP cells that were activated. (3) Solorzano et al.^14^ reported that only ∼70% of neurons with GRP mRNA were EGFP^+^ in this mouse line and therefore EGFP-negative GRP-expressing neurons may have been activated. However, this explanation would require that there was a specific subpopulation of GRP-expressing neurons that lacked EGFP and that these were selectively activated by chloroquine.

Alternatively, the GRP-expressing dorsal horn interneurons may not be critically involved in itch pathways. There continues to be considerable debate about whether GRP is released from primary afferents,^12,24^ and if it is then this would be consistent with our findings. However, the majority view now appears to be against primary afferents as a source of GRP,^15–18^ at least in naïve animals.^14^

Finally, it is possible that the GRPR on lamina I neurons is activated by a different peptide. Although the other main bombesin-like peptide, neuromedin B, is expressed in primary afferents,^15–18^ it has a very low affinity for the GRPR^54^ and is therefore unlikely to mediate its activation following pruritic stimulation. However, there may be another, as yet unknown, peptide that forms the link between pruriceptive primary afferents and the GRPRs that are expressed by lamina I neurons, and the fact that there is an orphan receptor (BB3) would be consistent with this suggestion.^54^

## Conclusions

The results of this study show that both Fos and pERK can be used to identify cells that have been activated by intradermal injections of the pruritogen chloroquine and that similar numbers of cells are labelled for each marker. However, they do not support the suggestion that GRP-expressing interneurons in the superficial dorsal horn are preferentially activated by chloroquine.

## References

[1] Bautista DM Wilson SR Hoon MA Why we scratch an itch: the molecules, cells and circuits of itch. Nat Neurosci 2014; 17: 175–182. 24473265 10.1038/nn.3619 PMC4364402

[2] LaMotte RH Dong X Ringkamp M Sensory neurons and circuits mediating itch. Nat Rev Neurosci 2014; 15: 19–31. 24356071 10.1038/nrn3641 PMC4096293

[3] Ross SE Pain and itch: insights into the neural circuits of aversive somatosensation in health and disease. Curr Opin Neurobiol 2011; 21: 880–887. 22054924 10.1016/j.conb.2011.10.012

[4] Akiyama T Carstens E Neural processing of itch. Neuroscience 2013; 250: 697–714. 23891755 10.1016/j.neuroscience.2013.07.035 PMC3772667

[5] Davidson S Zhang X Yoon CH The itch-producing agents histamine and cowhage activate separate populations of primate spinothalamic tract neurons. J Neurosci 2007; 27: 10007–10014. 17855615 10.1523/JNEUROSCI.2862-07.2007 PMC3008349

[6] Moser HR Giesler GJ Jr Characterization of pruriceptive trigeminothalamic tract neurons in rats. J Neurophysiol 2014; 111: 1574–1589. 24478156 10.1152/jn.00668.2013 PMC4035772

[7] Roberson DP Gudes S Sprague JM Activity-dependent silencing reveals functionally distinct itch-generating sensory neurons. Nat Neurosci 2013; 16: 910–918. 23685721 10.1038/nn.3404 PMC3695070

[8] Sun YG Chen ZF A gastrin-releasing peptide receptor mediates the itch sensation in the spinal cord. Nature 2007; 448: 700–703. 17653196 10.1038/nature06029

[9] Sun YG Zhao ZQ Meng XL Cellular basis of itch sensation. Science 2009; 325: 1531–1534. 19661382 10.1126/science.1174868 PMC2786498

[10] Akiyama T Tominaga M Takamori K Roles of glutamate, substance P, and gastrin-releasing peptide as spinal neurotransmitters of histaminergic and nonhistaminergic itch. Pain 2014; 155: 80–92. 24041961 10.1016/j.pain.2013.09.011 PMC3947363

[11] Zhao ZQ Huo FQ Jeffry J Chronic itch development in sensory neurons requires BRAF signaling pathways. J Clin Invest 2013; 123: 4769–4780. 24216512 10.1172/JCI70528 PMC3809799

[12] Liu XY Wan L Huo FQ B-type natriuretic peptide is neither itch-specific nor functions upstream of the GRP-GRPR signaling pathway. Mol Pain 2014; 10: 4. 24438367 10.1186/1744-8069-10-4 PMC3930899

[13] Takanami K Sakamoto H Matsuda KI Distribution of gastrin-releasing peptide in the rat trigeminal and spinal somatosensory systems. J Comp Neurol 2014; 522: 1858–1873. 24254931 10.1002/cne.23506

[14] Solorzano C Villafuerte D Meda K Primary afferent and spinal cord expression of gastrin-releasing peptide: message, protein, and antibody concerns. J Neurosci 2015; 35: 648–657. 25589759 10.1523/JNEUROSCI.2955-14.2015 PMC4293415

[15] Fleming MS Ramos D Han SB The majority of dorsal spinal cord gastrin releasing peptide is synthesized locally whereas neuromedin B is highly expressed in pain- and itch-sensing somatosensory neurons. Mol Pain 2012; 8: 52. 22776446 10.1186/1744-8069-8-52 PMC3495671

[16] Goswami SC Thierry-Mieg D Thierry-Mieg J Itch-associated peptides: RNA-Seq and bioinformatic analysis of natriuretic precursor peptide B and gastrin releasing peptide in dorsal root and trigeminal ganglia, and the spinal cord. Mol Pain 2014; 10: 44. 25123163 10.1186/1744-8069-10-44 PMC4132360

[17] Usoskin D Furlan A Islam S Unbiased classification of sensory neuron types by large-scale single-cell RNA sequencing. Nat Neurosci 2015; 18: 145–153. 25420068 10.1038/nn.3881

[18] Wada E Way J Lebacq-Verheyden AM Neuromedin B and gastrin-releasing peptide mRNAs are differentially distributed in the rat nervous system. J Neurosci 1990; 10: 2917–2930. 2398368 10.1523/JNEUROSCI.10-09-02917.1990 PMC6570249

[19] Gutierrez-Mecinas M Watanabe M Todd AJ Expression of gastrin-releasing peptide by excitatory interneurons in the mouse superficial dorsal horn. Mol Pain 2014; 10: 79. 25496164 10.1186/1744-8069-10-79 PMC4320531

[20] Hokfelt T Kellerth JO Nilsson G Substance-P—localization in central nervous system and in some primary sensory neurons. Science 1975; 190: 889–890. 242075 10.1126/science.242075

[21] Todd AJ Spike RC The localization of classical transmitters and neuropeptides within neurons in laminae I-III of the mammalian spinal dorsal horn. Prog Neurobiol 1993; 41: 609–645. 7904359 10.1016/0301-0082(93)90045-t

[22] Wang X Zhang J Eberhart D Excitatory superficial dorsal horn interneurons are functionally heterogeneous and required for the full behavioral expression of pain and itch. Neuron 2013; 78: 312–324. 23622066 10.1016/j.neuron.2013.03.001 PMC3700415

[23] Allen Spinal Cord Atlas, http://mousespinal.brain-map.org/.

[24] Mishra SK Hoon MA The cells and circuitry for itch responses in mice. Science 2013; 340: 968–971. 23704570 10.1126/science.1233765 PMC3670709

[25] Gutierrez-Mecinas M, Furuta T, Watanabe M, et al. A quantitative study of neurochemically-defined excitatory interneuron populations in laminae I-III of the mouse spinal cord. *Mol Pain* 2016. DOI: 10.1177/1744806916629065. 10.1177/1744806916629065 PMC4946630 27030714

[26] Hoon MA Molecular dissection of itch. Curr Opin Neurobiol 2015; 34C: 61–66. 25700248 10.1016/j.conb.2015.01.017 PMC4539294

[27] Hunt SP Pini A Evan G Induction of c-fos-like protein in spinal cord neurons following sensory stimulation. Nature 1987; 328: 632–634. 3112583 10.1038/328632a0

[28] Akiyama T Curtis E Nguyen T Anatomical evidence of pruriceptive trigeminothalamic and trigeminoparabrachial projection neurons in mice. J Comp Neurol 2016; 524: 244–256. 26099199 10.1002/cne.23839 PMC4946801

[29] Nojima H Carstens MI Carstens E c-fos expression in superficial dorsal horn of cervical spinal cord associated with spontaneous scratching in rats with dry skin. Neurosci Lett 2003; 347: 62–64. 12865142 10.1016/s0304-3940(03)00609-8

[30] Han L Ma C Liu Q A subpopulation of nociceptors specifically linked to itch. Nat Neurosci 2013; 16: 174–182. 23263443 10.1038/nn.3289 PMC3557753

[31] Nakano T Andoh T Lee JB Different dorsal horn neurons responding to histamine and allergic itch stimuli. Neuroreport 2008; 19: 723–726. 18418246 10.1097/WNR.0b013e3282fdf6c5

[32] Yao GL Tohyama M Senba E Histamine-caused itch induces Fos-like immunoreactivity in dorsal horn neurons: effect of morphine pretreatment. Brain Res 1992; 599: 333–337. 1291036 10.1016/0006-8993(92)90409-3

[33] Ji RR Baba H Brenner GJ Nociceptive-specific activation of ERK in spinal neurons contributes to pain hypersensitivity. Nat Neurosci 1999; 2: 1114–1119. 10570489 10.1038/16040

[34] Zhang L Jiang GY Song NJ Extracellular signal-regulated kinase (ERK) activation is required for itch sensation in the spinal cord. Mol Brain 2014; 7: 25. 24708812 10.1186/1756-6606-7-25 PMC3986448

[35] Polgar E Sardella TC Tiong SY Functional differences between neurochemically defined populations of inhibitory interneurons in the rat spinal dorsal horn. Pain 2013; 154: 2606–2615. 23707280 10.1016/j.pain.2013.05.001 PMC3858808

[36] Guillery RW On counting and counting errors. J Comp Neurol 2002; 447: 1–7. 11967890 10.1002/cne.10221

[37] Cameron D Gutierrez-Mecinas M Gomez-Lima M The organisation of spinoparabrachial neurons in the mouse. Pain 2015; 156: 2061–2071. 26101837 10.1097/j.pain.0000000000000270 PMC4770364

[38] Polgár E Campbell AD MacIntyre LM Phosphorylation of ERK in neurokinin 1 receptor-expressing neurons in laminae III and IV of the rat spinal dorsal horn following noxious stimulation. Mol Pain 2007; 3: 4. 17309799 10.1186/1744-8069-3-4 PMC1803781

[39] Mullen RJ Buck CR Smith AM NeuN, a neuronal specific nuclear protein in vertebrates. Development 1992; 116: 201–211. 1483388 10.1242/dev.116.1.201

[40] Todd AJ Spike RC Polgar E A quantitative study of neurons which express neurokinin-1 or somatostatin sst2a receptor in rat spinal dorsal horn. Neuroscience 1998; 85: 459–473. 9622244 10.1016/s0306-4522(97)00669-6

[41] McDonald JH Handbook of biological statistics, 3rd ed Baltimore, MD: Sparky House Publishing, 2014.

[42] Takahashi Y Chiba T Kurokawa M Dermatomes and the central organization of dermatomes and body surface regions in the spinal cord dorsal horn in rats. J Comp Neurol 2003; 462: 29–41. 12761822 10.1002/cne.10669

[43] Akiyama T Curtis E Nguyen T Anatomical evidence of pruriceptive trigeminothalamic and trigeminoparabrachial projection neurons in mice. J Comp Neurol 2016; 524: 244–256. 26099199 10.1002/cne.23839 PMC4946801

[44] Akiyama T Merrill AW Zanotto K Scratching behavior and Fos expression in superficial dorsal horn elicited by protease-activated receptor agonists and other itch mediators in mice. J Pharmacol Exp Ther 2009; 329: 945–951. 19293390 10.1124/jpet.109.152256 PMC2683773

[45] Hamada R Seike M Kamijima R Neuronal conditions of spinal cord in dermatitis are improved by olopatadine. Eur J Pharmacol 2006; 547: 45–51. 16934247 10.1016/j.ejphar.2006.06.058

[46] Han N Zu JY Chai J Spinal bombesin-recognized neurones mediate more nonhistaminergic than histaminergic sensation of itch in mice. Clin Exp Dermatol 2012; 37: 290–295. 22329438 10.1111/j.1365-2230.2011.04314.x

[47] Inan S Dun NJ Cowan A Inhibitory effect of lidocaine on pain and itch using formalin-induced nociception and 5’-guanidinonaltrindole-induced scratching models in mice: behavioral and neuroanatomical evidence. Eur J Pharmacol 2009; 616: 141–146. 19549515 10.1016/j.ejphar.2009.06.026 PMC2735214

[48] Lieu T Jayaweera G Zhao P The bile acid receptor TGR5 activates the TRPA1 channel to induce itch in mice. Gastroenterology 2014; 147: 1417–1428. 25194674 10.1053/j.gastro.2014.08.042 PMC4821165

[49] Nojima H Cuellar JM Simons CT Spinal c-fos expression associated with spontaneous biting in a mouse model of dry skin pruritus. Neurosci Lett 2004; 361: 79–82. 15135898 10.1016/j.neulet.2003.12.013

[50] Nojima H Simons CT Cuellar JM Opioid modulation of scratching and spinal c-fos expression evoked by intradermal serotonin. J Neurosci 2003; 23: 10784–10790. 14645470 10.1523/JNEUROSCI.23-34-10784.2003 PMC6740982

[51] Kawasaki Y Kohno T Zhuang ZY Ionotropic and metabotropic receptors, protein kinase A, protein kinase C, and Src contribute to C-fiber-induced ERK activation and cAMP response element-binding protein phosphorylation in dorsal horn neurons, leading to central sensitization. J Neurosci 2004; 24: 8310–8321. 15385614 10.1523/JNEUROSCI.2396-04.2004 PMC6729681

[52] Akiyama T Nagamine M Carstens MI Behavioral model of itch, alloknesis, pain and allodynia in the lower hindlimb and correlative responses of lumbar dorsal horn neurons in the mouse. Neuroscience 2014; 266: 38–46. 24530451 10.1016/j.neuroscience.2014.02.005 PMC4063361

[53] Akiyama T Nguyen T Curtis E A central role for spinal dorsal horn neurons that express neurokinin-1 receptors in chronic itch. Pain 2015; 156: 1240–1246. 25830923 10.1097/j.pain.0000000000000172 PMC4474752

[54] Ramos-Alvarez I Moreno P Mantey SA Insights into bombesin receptors and ligands: highlighting recent advances. Peptides 2015; 72: 128–144. 25976083 10.1016/j.peptides.2015.04.026 PMC4641779

